# A prospective evaluation of diagnostic methodologies for the acute diagnosis of dengue virus infection on the Thailand-Myanmar border

**DOI:** 10.1016/j.trstmh.2010.09.007

**Published:** 2011-01

**Authors:** Wanitda Watthanaworawit, Paul Turner, Claudia L. Turner, Ampai Tanganuchitcharnchai, Richard G. Jarman, Stuart D. Blacksell, François H. Nosten

**Affiliations:** a Shoklo Malaria Research Unit (SMRU), 68/30 Ban Toong Road, Mae Sot, Tak 63110, Thailand; b Mahidol University-Oxford Tropical Medicine Research Unit (MORU), Faculty of Tropical Medicine, Mahidol University, 420/6 Rajvithi Road, Bangkok 10400, Thailand; c Centre for Tropical Medicine, University of Oxford, Churchill Hospital, Oxford OX3 7LJ, UK; d Armed Forces Research Institute of Medical Sciences (AFRIMS), Bangkok 10400, Thailand

**Keywords:** Dengue, PCR, Serology, Evaluation, Diagnosis, Thailand

## Abstract

Clinically useful diagnostic tests of dengue virus infection are lacking. We prospectively evaluated the performance of real-time reverse transcriptase (rRT)-PCR, NS-1 antigen and IgM antibody tests to confirm dengue virus infection in acute blood specimens from 162 patients presenting with undifferentiated febrile illness compatible with dengue infection. rRT-PCR was the most sensitive test (89%) and potentially could be used as a single test for confirmation of dengue infection. NS-1 antigen and IgM antibody were not sufficiently sensitive to be used as a single confirmatory test with sensitivities of 54% and 17% respectively. The specificities of rRT-PCR, NS-1 antigen and IgM antibody tests were 96%, 100% and 88% respectively. Combining NS-1 and rRT-PCR or the combination of all three tests resulted in the highest sensitivity (93%) but specificities dropped to 96% and 83% respectively. We conclude that at least the combination of two tests, either agent detection (rRT-PCR) or antigen detection (NS-1) plus IgM antibody detection should be used for laboratory confirmation of dengue infection.

## 1 Introduction

Dengue virus is the most important arboviral disease in humans, with an estimated 100 million cases of dengue fever (DF) and several hundred thousand cases of dengue haemorrhagic fever (DHF) each year.^1^ Cases of DF and DHF were increasingly reported in nine countries within the South East Asia region between 1985 and 2006, with Thailand reporting the highest number of cases in the region until 2003.^2^ In South East Asia, adults with dengue virus infection usually present with an acute, undifferentiated, febrile illness.^3^, ^4^, ^5^, ^6^, ^7^ Previous reports have documented the difficulty in clinically differentiating dengue from other causes of fever, including leptospirosis^8^ and scrub typhus.^5^ Given this difficulty, and the fact that delayed antimicrobial treatment for such infections may result in increased mortality, reliable and rapid dengue confirmatory tests are needed. Additionally, rapid confirmation of dengue infection would facilitate improved monitoring of confirmed cases for development of complications such as shock or haemorrhage.^9^

Accurate laboratory confirmation of dengue infection involves a combination of tests depending on timing of infection. During the acute phase of infection, virus culture, nucleic acid detection (RT-PCR)^10^, ^11^, ^12^ or antigen detection (for example, by NS-1 antigen ELISA^13^, ^14^, ^15^) may be used for diagnosis. Serology is also used to confirm infection and distinguish primary and secondary infections by determining the differences between IgM and IgG antibody response and is currently more widely used as one of the laboratory diagnostic methods.^16^ There are a variety of serological methods described, including ELISA and haemagglutination inhibition (HI) tests, some of which are commercially available.^17^, ^18^ However, serological diagnosis of dengue infection requires paired serum specimens, resulting in retrospective, rather than rapid and clinically useful, confirmation of infection.

In the present paper, we have prospectively evaluated three diagnostic methodologies (IgM antibody ELISA, NS-1 antigen ELISA, and real-time reverse transcriptase [rRT]-PCR) for the detection of dengue virus infection in patients presenting with undifferentiated fever in a dengue endemic area, with the aim of determining the optimal acute specimen testing strategy in a routine clinical setting. This is the first head-to-head assessment of PCR and a selection of widely-applied commercial serological assays (NS-1 antigen, IgM and IgG antibodies). It is also the first diagnostic assessment that has demonstrated the improved diagnostic accuracy when selections of PCR, NS-1 antigen, IgM and IgG antibody test results are combined.

## 2 Materials and Methods

### 2.1 Patients

Shoklo Malaria Research Unit (SMRU) undertakes surveillance for malaria and other infectious diseases, and provides general medical care for the migrant and refugee population, in five clinics in rural Thailand, on the Thailand-Myanmar (Burma) border. We conducted dengue surveillance from April to August 2008 in the SMRU clinics at Mawker Thai village (MKT) and Maela refugee camp (MLA), both located in rural Tak province approximately 500 km northwest of Bangkok. Adult patients (age ≥15 years old) presenting to the clinics with fever ≥38°C of less than seven days duration and any clinical symptoms or physical signs consistent with dengue (abnormal bleeding, eye redness, headache, myalgia or rash) were included in the surveillance and blood was drawn to inform clinical management (blood culture, complete blood count, and plasma for serology). Patients who had a clear alternative diagnosis such as malaria, urinary tract infection or pneumonia were excluded from the surveillance.

In addition to dengue virus, patients were serologically investigated for leptospirosis (ELISA with confirmation by the microscopic agglutination test) and rickettsial infection (ELISA with confirmation by indirect immunofluorescence assay), the other common causes of undifferentiated fever in SE Asia.

### 2.2 Plasma specimens

A 6 ml acute venous blood specimen was collected from each patient in a sterile EDTA tube (Becton Dickinson, Franklin Lakes, NJ, USA) for dengue rRT-PCR, NS-1 antigen detection, and dengue IgM and IgG antibody detection tests. The patients were reviewed at a 10–14 day follow-up visit and a repeat 6 ml convalescent venous blood specimen was collected for dengue IgM and IgG antibody tests. Plasma was separated by centrifugation and frozen at −80 °C prior to the assays.

### 2.3 RNA extraction and RT-PCR

Viral RNA was extracted from 150 μl of the acute plasma specimens using the NucleoSpin RNA virus extraction kit (Macherey Nagel, Düren, Germany) according to the manufacturer's instructions and was stored at −80 °C prior to testing. One-step SYBR Green-based rRT-PCR, modified from Shu et al., was performed using the RotorGene 6000 real-time PCR system (Corbett Research, San Francisco, CA, USA).^11^ The reaction volume was 25 μl, comprising 10 μl of extracted RNA, 1 μl of each primer (10 μM), 12.5 μl of 2X SYBR Green reaction mix (containing 0.4 mM of each dNTP and 6 mM MgSO_4_) and 0.5 μl of SuperScript III RT/Platinum *Taq* Mix (SuperScript III Platinum SYBR Green One-Step Quantitative RT-PCR Kit, Invitrogen, Carlsbad, CA, USA). The amplification conditions consisted of reverse transcription at 50 °C for 30 min, 95 °C for 5 min for *Taq* inhibitor inactivation, followed by 45 cycles of 95 °C for 10 s, 54 °C for 30 s and 72 °C for 30 s. Melting curve analysis was used to confirm the specificity of the amplicons. Positive (extracted RNA from control strains of DENV1-4) and negative controls were included in each PCR run and the run only accepted if all controls gave appropriate results.

The serotype of dengue virus was sought from the acute plasma specimen in all patients with serologically confirmed dengue infection using a nested RT-PCR assay,^12^ modified by the Armed Forces Research Institute of Medical Sciences (AFRIMS), Bangkok, Thailand.^19^

### 2.4 NS-1 antigen detection

The Panbio Dengue Early ELISA (cat. no. E-DEN01P, lot. no. 08140; Panbio, Brisbane, Queensland, Australia) was used to detect NS-1 antigen in the acute plasma specimens only, following the manufacturer's instructions. A positive specimen was defined as having >11 Panbio units; <9 Panbio units was defined as negative; and 9–11 Panbio units was equivocal and the specimen retested to confirm the result.

Panbio units were calculated by first determining the assay cut-off value: the lot specific calibration factor was multiplied by the average absorbance result of the kit calibrator (internal control, run in triplicate). Subsequently, an index value was calculated for each patient specimen by dividing the specimen absorbance result by the cut-off value. Finally the Panbio units were determined by multiplying the index value by 10.

### 2.5 Dengue IgM and IgG antibody capture ELISAs

We used the dengue IgM (Panbio: cat. no. E-DEN01 M, lot. no. 08316) and IgG (Panbio: cat. no. E-DEN02G, lot. no. 09080) antibody capture ELISAs to detect IgM and IgG antibodies in both the acute and convalescent plasma specimens, following the manufacturer's instructions. A positive specimen was defined as having >11 Panbio units for IgM and >22 for IgG antibodies; <9 and <18 Panbio units was defined as negative for IgM and IgG antibodies, respectively; 9–11 Panbio units was equivocal for IgM and 18–22 Panbio units was equivocal for IgG antibodies and the specimen retested to confirm the result. Panbio units were calculated as described above.

### 2.6 Classification of dengue infection status

Dengue infection was classified using the above described commercial serological assays as ‘confirmed’ acute dengue infection based on WHO dengue diagnostic criteria as defined in Table 1. ‘Confirmed’ acute dengue cases were those that demonstrated an IgM or IgG antibody sero-conversion based on paired serum collections.^16^ Patients with static IgM positivity (i.e., positive in both acute and convalescent specimens, but with no rise in Panbio units) were considered to have evidence of recent dengue infection.

**Table 1:** Interpretation algorithm for dengue virus serology using the Panbio Dengue Early ELISA, IgM and IgG antibody capture ELISA kits

Patients (*n* = 76)	Acute specimen			Convalescent specimen		Interpretations		
	[Median Panbio Units (IQR)]					Justification for ‘acute dengue’ interpretation	WHO criteria interpretation	Reference interpretation
	NS1 Ag ≥ 11 Positive	IgM ≥ 11 Positive	IgG ≥ 22 Positive	IgM ≥ 11 Positive	IgG ≥ 22 Positive			
4	1.5 (1.5-1.6)	30.1(23.5-33.8)	4.0 (3.2-6.8)	24.4 (20.9-27.5)	4.2 (3.2-7.0)	IgM positive but no increase	-	Recent Dengue Infection
3	29.0 (24.9-29.4)	2.1 (1.9-2.3)	1.2 (1.1-1.3)	47.7 (32.9-49.0)	16.0 (12.1-16.9)	NS1 Ag positive; IgM sero-conversion	Confirmed	Confirmed Acute Dengue Infection
23	2.4 (1.7-5.8)	3.7 (2.8-5.2)	2.1 (1.7-3.8)	34.5 (24.4 -47.0)	43.4 (38.0-49.7)	IgM & IgG sero-conversion		
6	3.1 (2.3-4.1)	4.0 (3.6-4.1)	6.0 (2.3-7.9)	7.6 (6.8-8.4)	45.3 (39.9-51.5)	IgG sero-conversion		
4	4.3 (4.0-5.4)	21.8 (16.5-29.5)	3.3 (1.3-5.3)	56.9 (54.2-57.3)	43.1 (42.6-44.4)	IgM pairs increase; IgG sero-conversion		
27	24.2 (16.1-37.2)	4.1 (2.8-6.5)	1.2 (1.0-1.8)	45.5 (35.1-51.8)	41.5 (39.8-46.8)	NS1 Ag positive; IgM & IgG sero-conversion		
1	41.4	4.8	23.7	12.1	40.5	NS1 Ag positive; IgG pairs increase		
8	23.4 (14.1-37.1)	21.8 (13.9-40.0)	3.3 (2.5-4.7)	46.0 (29.8-51.5)	37.2 (34.1-41.3)	NS1 Ag positive; IgM pairs increase; IgG sero-conversion		

### 2.7 Data Analysis

All statistical analyses were performed by using STATA/SE for Macintosh, version 10.1 (Stata Corporation, College Station, TX, USA). Continuous variables were compared using Wilcoxon rank sum test (since non-normally distributed). Characteristics of the tests were analysed in 2 x 2 tables using the ‘diagt’ routine.^20^ The McNemar test was used to compare sensitivities of the tests.

## 3 Results

### 3.1 Patients

Two hundred and twenty nine patients were examined. All patients met the WHO clinical case definition for acute dengue infection.^21^ One hundred and sixty two patients (71%) returned for the follow-up specimen collection visit and were considered further.

Of the 162 patients included in the current analysis, 72 patients (44%) were given a laboratory confirmed diagnosis of dengue infection by paired serology (Table 1). Four patients were found to have evidence of recent dengue infection. The demographic and clinical data of the patients are shown in Table 2. Patients with confirmed dengue infection had lower white cell counts (4.8 vs 7.2, *P*<0.0001) and platelets (147 vs. 162, *P*=0.03) than patients with non-dengue infection.

**Table 2:** Demographic and clinical data of patients (*n* = 162)

	Dengue cases (*n *= 72)	Non-dengue cases (*n *= 90)
Sex (male)	42/72 (58%)	56/90 (62%)
Age (years)	23 (range: 15–63)	27 (range: 15–60)
Headache	72/72 (100%)	86/90 (96%)
Arthralgia	55/72 (76%)	67/90 (74%)
Myalgia	44/72 (61%)	57/90 (63%)
Retro-orbital pain	46/72 (64%)	52/90 (58%)
Rash	4/72 (6%)	4/90 (4%)
Fever duration (days)	2 (range: 1–5)	2 (range: 1–6)
Presenting temperature (°C)	38.8 (IQR: 38.3–39.2)	38.4 (IQR: 38.0–38.9)
Haemoglobin (g/dL)	13.3 (IQR: 12.0–14.6)	13.7 (IQR: 12.1–14.4)
White cell count (x10^3^/mm^3^)	4.8 (IQR: 3.6–6.4)	7.2 (IQR: 5.2–9.7)
Platelets (x10^3^/mm^3^)	147 (IQR: 106–196)	162 (IQR: 133–212)

Twenty six patients (16%) were found to have non-dengue causes for their illness: four patients (2%) grew *Salmonella typhi* from blood cultures, by serology nine patients (6%) had murine typhus, seven (4%) had scrub typhus, and six (4%) had leptospirosis. Only one patient had evidence of dual infection: acute secondary dengue and typhoid; this patient had positive NS-1 antigen and dengue rRT-PCR and also grew *Salmonella typhi* from a blood culture.

The serotype of dengue virus was sought by performing nested RT-PCR on the acute specimens from the 72 serologically confirmed cases. Seventy one of the cases (99%) were serotype 3, with the remaining case serotype 2. The four patients with serological evidence of recent dengue infection were negative in the nested RT-PCR.

### 3.2 Comparison of the characteristics of the individual diagnostic tests

The performance characteristics of NS-1 antigen detection, rRT-PCR, and IgM antibody detection in the acute plasma specimen against our ‘gold standard’ of paired serology are shown in Table 3. NS-1 antigen or IgM antibody test alone had low sensitivity compared with the paired serology result, 54% and 17% respectively. rRT-PCR sensitivity was 89%, significantly higher than both NS-1 antigen and IgM antibody tests (*P*<0.00001). Specificities of NS-1 antigen detection, rRT-PCR, and IgM antibody detection were 100%, 96% and 88% respectively.

**Table 3:** Diagnostic accuracy of NS-1 antigen detection, rRT-PCR, and IgM antibody detection on acute plasma specimens

	Tests		Paired serology result		Sensitivity % (95% CI)	Specificity % (95% CI)	PPV % (95% CI)	NPV % (95% CI)
			Dengue	Not dengue				
Individual assays	NS-1	+	39	0	54.2 (42.0-66.0)	100 (96.0-100)	100 (91.0-100)	73.2 (64.4-80.8)
		−	33	90				
	rRT-PCR	+	64	4	88.9 (79.3-95.1)	95.6 (89.0-98.8)	94.1 (85.6-98.4)	91.5 (83.9-96.3)
		−	8	86				
	IgM	+	12	11	16.7 (8.9-27.3)	87.8 (79.2-93.7)	52.2 (30.6-73.2)	56.8 (48.2-65.2)
		−	60	79				
Combined assays	NS-1+rRT-PCR	+	67	4	93.1 (84.5-97.7)	95.6 (89.0-98.8)	94.4 (86.2-98.4)	94.5 (87.6-98.2)
		−	5	86				
	NS-1+IgM	+	43	11	59.7 (47.5-71.1)	87.8 (79.2-93.7)	79.6 (66.5-89.4)	73.1 (63.8-81.2)
		−	29	79				
	rRT-PCR+IgM	+	66	15	91.7 (82.7-96.9)	83.3 (74.0-90.4)	81.5 (71.3-89.2)	92.6 (84.6-97.2)
		−	6	75				
	NS-1+rRT-PCR+IgM	+	67	15	93.1 (84.5-97.7)	83.3 (74.0-90.4)	81.7 (71.6-89.4)	93.8 (86.0-97.9)
		−	5	75				

rRT: reverse transcriptase real-time; PPV: positive predictive value; NPV: negative predictive value.

**Figure 1:**
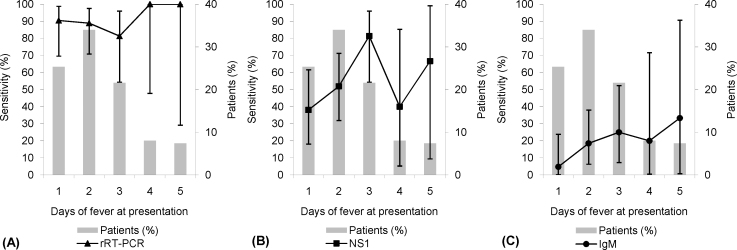
The effect of fever duration on test sensitivity: (A) rRT-PCR, (B) NS-1 antigen and (C) IgM antibody.

The effect of fever duration at presentation on assay sensitivity is shown in Figure 1. NS-1 antigen sensitivity peaked in the early stages of fever (three days of fever at presentation). IgM antibody sensitivity rose later, peaking in patients presenting with five days of fever. The sensitivity of rRT-PCR remained high throughout. However, as a result of the relatively small number of specimens on each day, particularly for four and five days of fever, the 95% confidence intervals around the sensitivities are wide.

### 3.3 Comparison of the characteristics of diagnostic test combinations

The performance characteristics of combinations of the acute specimen tests are shown in Table 3 (combined by an ‘OR’ operator). The overall sensitivity of rRT-PCR combined with IgM antibody detection (92%) was significantly better than IgM antibody detection alone (*P*<0.00001), but not significantly better than rRT-PCR alone (*P*=0.5). This was also true for the combination of IgM+NS-1+rRT-PCR (93%), which was significantly better than NS-1 antigen or IgM antibody detection alone (*P*<0.00001), but not better than rRT-PCR alone (*P*=0.2). The combination of NS-1+rRT-PCR was significantly more sensitive than NS-1 antigen detection alone (*P*<0.00001), but was not significantly better than rRT-PCR alone (*P*=0.2). NS-1+IgM was significantly more sensitive than IgM antibody alone (*P*<0.0001), but not significantly better than NS-1 antigen detection alone (*P*=0.1). Combining tests resulted in a fall in specificity. Combining rRT-PCR with IgM antibody alone or with IgM antibody and NS-1 antigen resulted in a specificity of 83%, whereas combining NS-1 antigen with rRT-PCR or IgM antibody, the specificities were 96% and 88% respectively.

## 4 Discussion

An accurate and rapid method for the diagnosis of dengue virus infection would facilitate optimal patient management. In resource-poor settings, diagnosis based on clinical features is the norm but, as shown in the current study, clinical diagnosis of dengue using WHO criteria is non-specific: only 72/162 (44%) of patients meeting the clinical case definition in the current study had laboratory confirmed dengue infection. Twenty six patients (16%) meeting the dengue clinical case definition had an alternative, and antibiotic-treatable, cause for their illness. Unfortunately, for a definitive laboratory diagnosis of dengue using current techniques often a combination of tests are required.^22^ In spite of this, many clinical laboratories continue to rely on a single assay to confirm dengue infection, which, as we have demonstrated, may lead to diagnostic inaccuracy. Additionally, many currently available rapid immunochromatographic tests (ICT), used in the field for clinical decision-making, suffer from poor performance characteristics.^19^

Antigen or molecular-based assays are attractive options for rapid diagnosis of dengue infection because they can potentially detect infection before an antibody response develops. Indeed, detection of dengue NS-1 antigen by ELISA allowed detection of infection prior to sero-conversion and could be detected in serum from the first day after onset of fever up to day nine of fever.^23^ Shu et al. confirmed the ability of PCR to detect dengue virus RNA between day one and day seven of fever.^11^

In the current study, NS-1 antigen and acute IgM antibody detection did not detect the majority of confirmed dengue cases. The positive predictive value (PPV) for NS-1 antigen detection was excellent (100%) but the negative predictive value (NPV) was poor at 73%, resulting in many patients with confirmed dengue being missed if the test were to be used alone. Acute IgM antibody detection had poor PPV and NPV (52% and 57% respectively), resulting in many misdiagnosed cases. rRT-PCR had the best operating characteristics (sensitivity 89%, specificity 96%, PPV 94%, NPV 92%) and would be potentially sufficient as a single assay for confirmation of dengue infection, since it allows for accurate confirmation or refuting of infection. The combinations of NS-1+rRT-PCR or NS-1+IgM+rRT-PCR resulted in the highest sensitivity (93%), although this was associated with an inevitable fall in specificity (96% and 83% respectively).

Compared to previous studies on NS-1 antigen ELISA we report a slightly lower sensitivity. Dussart et al. found the Panbio NS-1 antigen ELISA to have a sensitivity of 60% when used on stored serum specimens from French Guiana^14^ and, in a similar study from Puerto Rico, Bessoff et al. reported a sensitivity of 65%.^13^ On prospectively collected specimens from clinically suspected dengue cases in Laos, Blacksell et al. reported a sensitivity of 63%.^24^ The sensitivity of rRT-PCR was slightly better than reported by the original authors who found that PCR detected viral RNA 83% of acute specimens from patients with confirmed dengue.^11^ Comparing operating characteristics of assays between studies can be difficult, since there are many potential confounding factors. Firstly, in the current study, specimens were collected prospectively on patients with illness broadly compatible with dengue whereas several of the previous evaluations of NS-1 antigen ELISA have been retrospective, using well characterised serum specimen collections. We feel that the results presented here are likely to more accurately reflect the operating characteristics of the tests in a routine clinical setting. Secondly, infections due to dengue serotype 3 predominated in our study, and previous work has noted that the Panbio NS-1 antigen ELISA may miss infections caused by this serotype.^24^ Thirdly, timing of presentation and specimen collection may affect assay performance: in our study, most patients presented very early in the course of their infection. Although we demonstrated trends in the sensitivity of each assay, the small number of patients presenting with more than three days of fever limited our ability to perform statistical analysis. Previous studies have demonstrated the effect of timing of presentation on NS-1 antigen and IgM antibody^24^ or PCR^11^ assays, but no comparison between antigen detection, PCR, and serology on the same patient population has been described. Finally, infection status (primary infection versus secondary infection) may also make study-to-study comparisons difficult. We identified very few patients with acute primary infection (3/72, using Panbio kit criteria), resulting in an inability to determine potential differences in test characteristics between primary and secondary infections. We plan to perform further work to delineate the optimum sampling ‘window’ for each assay for patients with primary and secondary dengue infection.

This work suggests that, in a clinical setting, combined tests (IgM antibody ELISA, NS-1 antigen ELISA and/or rRT-PCR) are still required to confidently diagnose acute dengue infection from a single blood specimen: at least a combination of two tests, either agent detection (rRT-PCR) or antigen detection (NS-1) plus IgM antibody should be used. Unfortunately, this results in slow laboratory confirmation of dengue infection. Given that rRT-PCR was the most accurate single assay in our assessment, prospective evaluations of new field-deployable rapid diagnostic molecular tests, for example LAMP-based assays, are planned. These evaluations will include comparisons with the newer combined NS-1 antigen and IgM antibody ICT rapid test kits. Although cheap and/or field-deployable PCR systems are some way off at the current time, progress has been made in this area and development and refinement of current techniques may result in nucleic acid detection becoming the standard for rapid dengue diagnosis even in resource-poor settings.^25^

## Funding

This work was supported by the Wellcome Trust (Grant no. 077166/Z/05).

## Conflicts of interest

None declared.

## Ethical approval

Not required.

Authors’ statement: The opinions or assertions contained herein are the private views of the authors, and not to be construed as official, or as reflecting true views of the Department of the Army or the Department of Defense.

## Authors’ contributions

FHN, CLT and PT conceived the work; CLT was responsible for the clinical work and specimen collection; AT and WW conducted and interpreted the laboratory work (serology and real-time PCR) under the supervision of SDB and PT; RGJ interpreted and analysed the dengue serotyping PCR results; SDB, PT and WW performed the final data analysis. WW prepared the first draft and all authors contributed to the revision of the manuscript and read and approved the final version. WW and FHN are guarantors of the paper.
